# Utility scores for different health states related to depression: individual participant data analysis

**DOI:** 10.1007/s11136-017-1536-2

**Published:** 2017-03-04

**Authors:** Spyros Kolovos, Judith E. Bosmans, Johanna M. van Dongen, Birre van Esveld, Dorcas Magai, Annemieke van Straten, Christina van der Feltz-Cornelis, Kirsten M. van Steenbergen-Weijenburg, Klaas M. Huijbregts, Harm van Marwijk, Heleen Riper, Maurits W. van Tulder

**Affiliations:** 1 Department of Health Sciences, and the EMGO Institute for Health and Care Research, Faculty of Earth and Life Sciences, VU University Amsterdam, De Boelelaan 1085, 1081 HV Amsterdam, The Netherlands; 2 Department of Clinical, Neuro and Developmental Psychology, and the EMGO Institute for Health and Care Research, Faculty of Behavioural and Movement Sciences, VU University Amsterdam, Amsterdam, The Netherlands; 3 Department of Social Psychiatry Tranzo, Tilburg University, Tilburg, The Netherlands; 4 Clinical Centre of Excellence for Body, Mind and Health, GGz Breburg, Tilburg, The Netherlands; 5 Dr. Leo Kannerhuis, Doorwerth, The Netherlands; 6 GGNet, Mental Health - RGC Winterswijk, Winterswijk, The Netherlands; 7 Centre for Primary Care, Institute for Population Health, University of Manchester, Manchester, UK; 8 Department of General Practice and Elderly Care Medicine, VU University Medical Centre, Amsterdam, The Netherlands

**Keywords:** Utility scores, Quality-of-life, Depression, Multilevel analysis, EQ-5D, SF-6D

## Abstract

**Objectives:**

Depression is associated with considerable impairments in health-related quality-of-life. However, the relationship between different health states related to depression severity and utility scores is unclear. The aim of this study was to evaluate whether utility scores are different for various health states related to depression severity.

**Methods:**

We gathered individual participant data from ten randomized controlled trials evaluating depression treatments. The UK EQ-5D and SF-6D tariffs were used to generate utility scores. We defined five health states that were proposed from American Psychiatric Association and National Institute for Clinical Excellence guidelines: remission, minor depression, mild depression, moderate depression, and severe depression. We performed multilevel linear regression analysis.

**Results:**

We included 1629 participants in the analyses. The average EQ-5D utility scores for the five health states were 0.70 (95% CI 0.67–0.73) for remission, 0.62 (95% CI 0.58–0.65) for minor depression, 0.57 (95% CI 0.54–0.61) for mild depression, 0.52 (95%CI 0.49–0.56) for moderate depression, and 0.39 (95% CI 0.35–0.43) for severe depression. In comparison with the EQ-5D, the utility scores based on the SF-6D were similar for remission (EQ-5D = 0.70 vs. SF-6D = 0.69), but higher for severe depression (EQ-5D = 0.39 vs. SF-6D = 0.55).

**Conclusions:**

We observed statistically significant differences in utility scores between depression health states. Individuals with less severe depressive symptoms had on average statistically significant higher utility scores than individuals suffering from more severe depressive symptomatology. In the present study, EQ-5D had a larger range of values as compared to SF-6D.

**Electronic supplementary material:**

The online version of this article (doi: 10.1007/s11136-017-1536-2) contains supplementary material, which is available to authorized users.

## Introduction

Depression is a common mental disorder with a 12-month prevalence of 5.3% and a lifetime prevalence of 13.2% [1]. It is expected to rank first in terms of disability-adjusted life-years (DALYs) in high-income countries by 2030 [1–3]. Depression constitutes an enormous societal cost due to increased absenteeism, decreased work performance, and high healthcare utilization of depressed individuals [4, 5]. Moreover, it is related to considerable reductions in health-related quality-of-life (HRQoL) [6, 7]. Impairments in HRQoL are seen in mental, physical, and social functioning and negatively affect various aspects of the individual’s daily life [8].

HRQoL can be expressed as a utility score that represents the relative societal desirability of a particular health state that is anchored by 0 (death) and 1 (perfect health) [9]. Utility scores are most commonly estimated using an indirect method, meaning that participants fill out a HRQoL questionnaire and then an algorithm is used to convert the participant’s health state into a utility score [10, 11]. These utility scores are often used to generate Quality-Adjusted Life-Years (QALYs) [10]. A QALY is a measure that combines quality and quantity of life lived and is calculated by multiplying the utility score by the amount of time a participant spent in a particular health state. Many national guidelines for economic evaluations, for example in the Netherlands and the United Kingdom, recommend using QALYs in economic evaluations because they allow for comparisons across different treatments and health problems [11].

Depression severity can be categorized in different health states, namely remission and minor, mild, moderate, and severe depression. The association between health states related to depression severity and utility scores is not yet well researched. Two studies have examined the relationship between utility scores and different health states [12, 13]. They showed that depression has a considerable impact on utility scores, where more severe depression was associated with lower utility scores.

However, the aforementioned studies have several limitations. First, participants in both studies received antidepressant medication [12, 13]. Consequently, the findings may not be generalizable to individuals with depressive symptoms receiving no treatment or other types of treatment such as psychotherapy or combined treatments. Secondly, relatively small sample sizes were used in the analyses (*n* = 70 and *n* = 447, respectively) [12, 13]. Thus, these two studies could be underpowered to detect small but statistically significant differences in utility scores between health states. Furthermore, one study used the EuroQoL- 5 Dimensions (EQ-5D) [14] to estimate utility scores and the other the Short-Form (SF)- 6 Dimensions [15]. However, it has been demonstrated that there are discrepancies between utility scores derived from the EQ-5D and the SF-6D leading to higher EQ-5D utility scores for healthier groups and higher SF-6D utility scores for less healthy groups [16, 17].

A recent meta-analysis aimed to pool the utility scores from different studies for three depression states (i.e., mild, moderate, and severe) [18]. In total, the results from three studies were pooled regarding EQ-5D utilities. The authors indicated that milder depressive symptoms were related to increased utility scores [18]. However, the number of included studies was limited and the statistical heterogeneity was considerable. Thus, the results may have limited generalizability and their validity may not be established.

Given the above, we included a large, representative sample of participants with depression, receiving interventions or being in control groups. Subsequently, we aimed to establish the utility scores, generated separately from EQ-5D and SF-6D tariffs for different health states related to depression severity. Secondly, we aimed to compare utility scores between clinically relevant depression health states. We hypothesized that the more severe health states would be related to lower utility scores and vice versa. The final objective was to investigate for potential differences between EQ-5D and SF-6D utility scores. We hypothesized that EQ-5D utility scores will have a wider range of values compared to the SF-6D [16, 17].

## Methods

### Study selection

We carried out an individual participant data meta-analysis to estimate utility scores per predefined health state and to compare utility scores between health states. We performed a search in PubMed to identify relevant studies, using terms indicative for depressive symptoms, treatment for depression, quality-of-life, and randomized controlled trials. We did not aim to conduct a systematic review because this was not necessary for answering our research questions.

Two researchers examined the eligibility of the identified studies (SK and JEB). RCTs were eligible if they (a) included participants with a diagnosis of a depressive disorder based on a structured clinical interview, or participants with elevated depressive symptomatology based on a standardized measure of depressive symptom severity, (b) compared a treatment for depression with a control condition (i.e., care as usual or a waiting list group), (c) administered the EQ-5D-3L and/or SF-12 or SF-36 as a measure of HRQoL, (d) included a measure of depressive symptom severity (e.g., PHQ-9), and (e) were conducted in the Netherlands (to facilitate data sharing).

### Data extraction and preparation

We contacted the authors of RCTs that satisfied our inclusion criteria and asked them permission to access their primary datasets. The authors signed a data sharing agreement that we provided. Data concerning participant registration number, gender, age, relationship status (not married/divorced/widowed, or married/living together with a partner), treatment group (intervention or control), education level (low, medium, high), comorbidity (study included exclusively participants with depression and another comorbid condition), and HRQoL and depression severity scores for all available measurements (i.e., baseline and follow-ups) were requested from the authors. All acquired data were strictly anonymous and it was not possible to track the identity of any of the participants. After receiving the primary datasets, we combined them in one database. Two researchers extracted the data from the primary datasets independently (SK and BvE or DM).

Utility scores were calculated using the UK EQ-5D and SF-6D tariffs (there are no Dutch tariffs available for SF-6D) [19, 20]. Included studies used different measures to monitor depressive symptom severity (Table 1). We used cut-off scores obtained from the literature for each of these measures to define the participant’s health state. The cut-off scores and the range of the questionnaires are reported in detail in Table 1. In accordance with the American Psychiatric Association (APA) [21] and National Institute for Clinical Excellence (NICE) [22] guidelines, we differentiated between five health states: remission (no or minimal depressive symptoms and no specific concern for clinical depression), minor depression (subthreshold/subclinical depression), and mild depression, moderate depression, and severe depression (three different severity levels of clinical depression).

**Table 1:** Cut-off scores for health states related to depressive symptom severity

Measures	Remission	Minor depressive symptoms	Mild depression	Moderate depression	Severe depression
PHQ-9 [50–52]	0–4	5–9	10–14	15–19	20–27
MADRS [53, 54]	0–8	9–18	19–26	27–34	35–60
CES-D [55–57]	0–15	16–19	20–25	26–30	31–60
IDS-SR [58, 59]	0–13	14–25	26–38	39–48	49–84
HADS-D [60, 61]	0–7	8–13	14–19	20–25	26–52

*CES-D* center for epidemiologic studies depression scale, *HADS-D* hospital anxiety and depression scale, *IDS-SR inventory of depressive symptomatology self-report; MADRS* Montgomery–Åsberg depression rating scale, *PHQ-9* patient health questionnaire

### Statistical analysis

We performed the analyses using the combined database. We used descriptive statistics to describe the demographic characteristics of the participants. To estimate the utility scores for each health state, we used a multilevel linear regression model in which we accounted for observations nested within participants and participants nested within studies (i.e., three-level structure). We used the default maximum-likelihood approach implemented in MLwiN [23]. Separate analyses were carried out for the EQ-5D and SF-6D. The utility scores were the dependent variables, and four dummy variables representing the five health states were the independent variables. Based on the literature, we added the variables comorbidity, gender, age, relationship status, randomization group, and education level to the model to examine possible confounding effects. To determine whether there was confounding, we used the ‘rule of thumb’ of 10% change in the random coefficients between the model without covariates (crude model) and the model with covariates (adjusted model) [24, 25]. We also carried out a linear regression analysis without taking into account the hierarchical structure of the data (‘baseline model’). Statistical significance was set at *p* < .05.

### Sensitivity analysis

We performed two sensitivity analyses. First, we repeated the analyses using the Dutch EQ-5D tariffs (there are no Dutch tariffs for SF-6D) [26], because we wanted to investigate whether our conclusions remain the same when using population preference values from different countries [27, 28]. In the second sensitivity analysis, we included only the baseline measurements from EQ-5D and SF-36 to calculate the mean utility scores for each health state. The main analyses included all the measurements of the participants (i.e., baseline and follow-ups) and, even though we controlled for this in the multilevel analysis, it is possible that it could influence our estimates.

## Results

### Characteristics of included studies

We included ten studies with 1629 participants. All studies were conducted in the Netherlands and are presented in detail in Table 2. Four of them evaluated psychological treatments as an intervention (i.e., interpersonal psychotherapy, problem solving treatment, and cognitive behavioral therapy), two evaluated collaborative care (i.e., combination of general practitioner, psychiatrist, psychotherapist, and depression care manager), two stepped care (i.e., watchful waiting, activity scheduling, life review and consultation, and general practitioner), one disease management (i.e., general practitioner screening and consultation), and one medication and care as usual (i.e., antidepressants, consultation and information on depression). As a comparator, eight studies included care as usual and two used waiting list groups. Four studies included participants with depression and another comorbid condition.

**Table 2:** Characteristics of the included studies

First author, year	Target group	Intervention	Control	Depression measure	HRQoL measure	Follow-Up^a^
Bosmans 2007 [62]	Primary care elder participants with major depression	Interpersonal psychotherapy = 69	CAU = 74	MADRS	EQ-5D-3L SF-36	26; 52
Bosmans 2014 [63]	Elder participants in elderly homes at risk for depressive and/or anxiety disorders	Stepped care prevention program = 93	CAU = 92	CES-D	EQ-5D-3L	4; 17; 30; 43
Hermens 2007 [64]	Primary care participants with minor or mild major depression	CAU + antidepressants = 85	CAU = 96	MADRS	EQ-5D-3L SF-36	6; 13; 52
Huijbregts 2013 [65]	Primary care participants with major depression	Collaborative care = 61	CAU = 38	PHQ-9	EQ-5D-3L	13; 26; 39; 52
van Marwijk 2008 [66]	Primary care participants with major depression	Program-based disease management = 70	CAU = 75	MADRS	EQ-5D-3L SF-36	9; 26; 52
Schreuders 2007 [67]	Primary care participants with mental health problems	PST + CAU = 88	CAU = 87	HADS-D	EQ-5D-3L SF-36	13; 39
Seekles 2011 [68]	Primary care participants with minor/major depression and anxiety disorder	Stepped care = 60	CAU = 60	IDS	EQ-5D-3L	8; 16; 24
van Steenbergen-Weijenburg 2015 [69]	Participants at general hospital with chronic diseases and major depression	Collaborative Care = 42	CAU = 40	PHQ-9	EQ-5D-3L SF-36	26; 52; 78; 104
van Straten 2007 [70]	Self-referred participants with depression, anxiety, or work-related stress	Web-based PST = 107	WL = 106	CES-D	EQ-5D-3L	5; 9
Warmerdam 2010 [71]	Self-referred participants with depressive symptoms	Web-based PST = 88; Web-based CBT = 88	WL = 87	CES-D	EQ-5D-3L	5; 8; 12; 39

*CAU* care as usual, *CBT* cognitive behavioral therapy, *CES-D* center for epidemiologic studies depression scale, *HADS-D* hospital anxiety and depression scale, *HRQoL* health-related quality-of-life, *IDS-SR* inventory of depressive symptomatology self-report, *MADRS* Montgomery–Åsberg depression rating scale, *PHQ-9* patient health questionnaire, *PST* problem solving treatment, *WL* Waiting list

^a^Follow-up in weeks from baseline

Depression measures included Center for Epidemiologic Studies Depression Scale (CES-D, *n* = 3), Montgomery–Åsberg Depression Rating Scale (MADRS, *n* = 3), Patient Health Questionnaire (PHQ-9, *n* = 2), Inventory of Depressive Symptomatology Self-Report (IDS-SR, *n* = 1), and Hospital Anxiety and Depression Scale (HADS-D, *n* = 1). All studies administered the EQ-5D-3L and five of them also administered the SF-36.

### Characteristics of participants

The demographic characteristics of the participants are presented in Table 3. From the 1629 participants, 856 had been randomized to an intervention group and 773 to a control group. Furthermore, 1087 participants were female (67%). The mean age was 56 years (SD = 18) and 720 participants (49%) were married or lived together with a partner (Table 3). Also, 569 participants (35%) had a lower education level (basic education or elementary school), 488 (31%) had an intermediate education level (high school or 12 years of education), and 536 (34%) had a higher education level (education after high school or university level degree).

**Table 3:** Demographic characteristics of participants*

Characteristic	Intervention (*n* = 856)	Control (*n* = 773)	Total (*n* = 1629)
Female	588 (69)	499 (65)	1087 (67)
Mean age (SD)	55 (18)	56 (18)	56 (18)
Relationship status			
Unmarried/ divorced/ widowed	379 (49)	383 (54)	762 (51)
Married/living together	388 (51)	332 (46)	720 (49)
Education level			
Low	284 (34)	285 (38)	569 (35)
Middle	254 (30)	234 (31)	488 (31)
High	301 (36)	235 (31)	536 (34)

Frequencies do not add up to *n* = 1629 due to missing data

*Presented are frequencies and valid percentages, unless otherwise indicated.

*SD* standard deviation

### EQ-5D utility scores

We included 4979 observations in the analyses. Table 4 presents the average utility scores from the adjusted model based on the EQ-5D and the mean differences between the health states. The average utility scores in the adjusted model were 0.70 (95% CI 0.67–0.73) for remission, 0.62 (95% CI 0.58–0.65) for minor depression, 0.57 (95% CI 0.54–0.61) for mild depression, 0.52 (95% CI 0.49–0.56) for moderate depression, and 0.39 (95%CI 0.35–0.43) for severe depression.

**Table 4:** Mean utility scores and mean differences (95% confidence intervals) for different health states of depression

Reference\comparator	Remission	Minor depression	Mild depression	Moderate depression	Severe depression
EQ-5D (*N*_obs_ = 4979)					
Remission	0.70 (0.67 to 0.73)	−0.08 (−0.10 to − 0.06)***	−0.13 (−0.15 to − 0.1.)***	−0.18 (− 0.20 to − 0.15)***	−0.34 (−0.37 to − 0.30) ***
Minor depression	X	0.62 (0.58 to 0.65)	−0.04 (−0.07 to −0.02)***	-0.09 (− 0.12 to − 0.07)***	−0.26 (−0.29 to − 0.22)***
Mild depression	X	X	0.57 (0.54 to 0.61)	−0.05 (− 0.07 to − 0.03)***	−0.21 (−0.25 to − 0.18)***
Moderate depression	X	X	X	0.52 (0.49 to 0.56)	−0.16 (−0.20 to −0.13)***
Severe depression	X	X	X	X	0.39 (0.35–0.43)
SF-6D (*N*_obs_ = 1726)					
Remission	0.69 (0.67–0.71)	−0.06 (−0.08 to −0.04)***	−0.10 (−0.12 to − 0.08)***	−0.13 (− 0.15 to − 0.11)***	−0.14 (−0.16 to − 0.12)***
Minor depression	X	0.63 (0.61 to 0.66)	−0.04 (−0.06 to 0.02)***	−0.07 (− 0.09 to − 0.05)***	−0.08 (− 0.10 to − 0.6)***
Mild depression	X	X	0.59 (0.58 to 0.61)	−0.03 (−0.05 to − 0.01)**	−0.04 (−0.06 to − 0.02)***
Moderate depression	X	X	X	0.56 (0.54 to 0.59)	−0.01 (−0.03 to 0.00)
Severe depression	X	X	X	X	0.55 (0.53 to 0.57)

*N*_*obs*_ number of observations

*P < .05; **P < .01; ***P < .001

The mean utility scores were statistically significantly different between all five health states. The largest mean difference was found between remission and severe depression (−0.34, 95% CI −0.37 to −0.30). The smallest mean difference was found between minor and mild depression (−0.04, 95% CI −0.07 to −0.02). The covariates including comorbidity, age, gender, relationship status, randomization group, and education level were included in the adjusted model but the random coefficients of health states did not change by more than 10% (see Supplementary material, Table S1). Thus, we inferred that these covariates did not confound our estimations.

### SF-6D utility scores

We used SF-6D utility scores as the dependent variable and included 1726 observations (Table 4). The average utility scores in the adjusted model were 0.69 (95% CI 0.67–0.71) for remission, 0.63 (95% CI 0.61–0.66) for minor depression, 0.59 (95% CI 0.58–0.62) for mild depression, 0.56 (95% CI 0.54–0.59) for moderate depression, and 0.55 (95% CI 0.53–0.57) for severe depression.

The mean differences in utility scores between the health states were statistically significant, except for the difference between moderate and severe depression (−0.01, 95% CI −0.03–0.00). We did not detect any confounding of our estimations (see Supplementary material, Table S1).

### Differences between EQ-5D and SF-6D utility scores

Overall, the mean differences in utility scores between the health states were larger for EQ-5D than for SF-6D. The SF-6D in comparison with the EQ-5D showed a smaller range of utility scores (Table 4). In particular, it generated slightly lower utility scores for participants in less severe health states, and higher scores for more severe health states such as severe depression (EQ-5D = 0.39 vs. SF-6D = 0.55) (Table 4).

### Sensitivity analyses

We calculated the utility scores using the Dutch EQ-5D tariffs. The average utility scores for the adjusted model were 0.73 (95% CI 0.69–0.77) for remission, 0.63 (95% CI 0.59–0.67) for minor depression, 0.58 (95% CI 0.54–0.62) for mild depression, 0.51 (95% CI 0.47–0.55) for moderate depression, and 0.37 (95%CI 0.33–0.41) for severe depression.

The sensitivity analysis using only baseline scores for EQ-5D (UK tariffs) included 1453 observations. The mean utility scores of the adjusted model were 0.72 (95% CI 0.63–0.80) for remission, 0.62 (95% CI 0.55–0.69) for minor depression, 0.55 (95% CI 0.47–0.62) for mild depression, 0.47 (95% CI 0.40–0.55) for moderate depression, and 0.30 (95% CI 0.23–0.38) for severe depression.

The sensitivity analysis using only baseline scores for SF-6D included 520 observations. The average utility scores of the adjusted model were 0.68 (95% CI 0.65–0.72) for remission, 0.63 (95% CI 0.60–0.63) for minor depression, 0.57 (95% CI 0.54–0.60) for mild depression, 0.55 (95% CI 0.52–0.58) for moderate depression, and 0.54 (95% CI 0.49–0.58) for severe depression.

## Discussion

The present study estimated utility scores derived from the EQ-5D and the SF-6D for five health states related to depression severity using individual participant data from ten clinical trials. The results demonstrated that utility scores differed statistically significant between the health states, and that less severe health states were associated with higher utility scores.

There are some differences between the utility scores that we found for each health state and those reported in the literature. For instance, the average utility score for remission (0.69–0.71) was somewhat lower than those reported in other studies (0.72–0.86) [12, 13]. In addition, the average utility score for severe depression in our study (0.39–0.55) was higher as compared to the previous findings (0.27–0.30) [12, 13]. These differences may be explained by the differences in design and methodology between the present and the previous studies. To illustrate, one of the previous studies evaluated depression severity based on physician’s judgment in combination with the Clinical Global Impression Improvement Scale (CGI-I) [13], while in the other study the participants evaluated hypothetical health states related to depression severity [12]. Furthermore, these studies were conducted in the US (using SF-6D) and Sweden (using EQ-5D) and used different tariffs to calculate utility scores.

The average utility score for remission found in our study was lower than the average utility score of the general population (between 0.76 and 0.87) [17, 29, 30]. This finding is in line with the literature, indicating that individuals in remission from depression may suffer from residual impairments in HRQoL [31, 32]. Thus, evaluating treatment success based on remission of depression symptoms alone may be too restrictive. Improvements in HRQoL may take longer to occur and should, therefore, be monitored after remission of depressive symptoms as well [31, 33].

There is doubt in the literature regarding the performance of EQ-5D and SF-6D in detecting small but important changes in utility values [34–36]. Although we showed that most of the differences in utility scores between the five health states were statistically significant, it is important to examine whether the detected differences are also clinically relevant [37]. Clinical relevance can be defined as the minimum change in an outcome that is perceived by the individuals as relevant and beneficial and has a notable effect on their daily life [38]. A previous systematic review showed that a clinically relevant change in utility score for the EQ-5D ranged between 0.01 and 0.14 (mean = 0.07) and for the SF-6D between 0.01 and 0.10 (mean = 0.04) [39]. Therefore, most of the mean differences in EQ-5D and SF-6D utility scores between the health states in our study appeared clinically relevant. There was no evidence that the differences between minor and mild, and mild and moderate depression for EQ-5D utility scores were clinically relevant. Similarly, for SF-6D utility scores, the differences between mild and moderate, and moderate and severe depression did not appear clinically relevant.

Our results are in line with the previous findings indicating that the EQ-5D generates higher utility scores than the SF-6D among healthier participants and lower scores for less healthier participants (i.e., severe depression) [17, 40]. This discrepancy has been addressed before [36, 41–43] and has been attributed to the different scoring algorithms, number of possible health states, and the approach through which the utility scores are generated [41]. It is, therefore, important to consider that utility scores for health states related to depression severity are dependent on the measure from which they are generated.

The sensitivity analyses that we conducted demonstrated the robustness of our results. In particular, when we used the Dutch EQ-5D tariffs, the mean utility scores changed slightly, as it was expected. Nevertheless, in line with the main analyses, participants with more severe depressive symptoms had on average lower utility scores. Similarly, when we included only the baseline measurements in our models, the mean utility scores were in accordance with those in the main analyses.

### Strengths and limitations

To our knowledge, this is the first study examining the relationship between utility scores and health states related to depression that includes a large sample size providing sufficient statistical power. Furthermore, we performed the analyses using multilevel modeling, which is considered the most appropriate approach to analyze hierarchically structured data and takes into account potential differences between the included studies [44]. We used utility scores generated from both the EQ-5D-3L and the SF-36. Finally, we provided mean utility scores for the five health states related to depression severity that are recommended by APA and NICE [21, 22].

In model-based economic evaluations, health economic models are used to examine the long-term cost-utility of interventions for depression. Utility scores are typically included in these models to calculate QALYs. Previous model-based studies populated their models with utility scores selected from only a limited set of studies [45]. Our study shows some important advantages over previous studies [12, 13] (e.g., large sample size, health states based on national guidelines) and our findings can be used to populate health economic models with more confidence. For instance, we intend to use the results of this study to populate a health economic model examining the cost-effectiveness of a “blended” (face-to-face and Internet-based) treatment for depression, which is part of the E-COMPARED project funded under the Seventh Framework Program [46].

The present study is not without limitations. We used different measures of depressive symptom severity to define the health states related to depression severity, while cut-off scores for depression were based on the literature [47]. However, some studies in the literature reported different cut-off scores for the same instruments. The combination of different measures and the employment of cut-off scores could potentially lead to overlapping health states. Nevertheless, as reflected by the clinically relevant and statistically significant mean differences in the utility scores, the health states were a reliable representation of depressive symptom severity.

We used tariffs generated from a UK population to calculate the utility scores. It is possible that utility scores would be slightly different if we would have used studies and population preference values from other countries [27, 28]. However, the sensitivity analyses using Dutch tariffs for EQ-5D showed that our findings are robust. Similarly, using the EQ-5D-5L, which was recently introduced, may result in different outcomes [48]. Furthermore, the mean age of our sample was somewhat high (i.e., 56 years old). Older age can be a factor related to lower HRQoL, but we statistically controlled for it without finding any statistically significant associations. In addition, comorbidity is very common for patients with depression and it may be related to impairments in HRQoL [49, 50]. We statistically controlled for comorbidity but we did not find any significant relationship. Finally, comorbidity and age of participants do not seem a threat to the validity of our estimations because the mean utility scores were similar or higher than utility scores reported for participants with depression in other studies.

## Conclusion

We demonstrated that there are statistically significant and clinically relevant differences in utility scores between the health states. Particularly, individuals with less severe depressive symptoms had on average higher utility scores than individuals with more severe depressive symptomatology. Considering that individuals in remission from depression had on average lower utility scores than the general population, it is important to take into account HRQoL as an outcome of depression treatments. Differences between EQ-5D and SF-6D utility scores, and particularly the larger range of EQ-5D values, need to be considered for future economic evaluations and health economic models.

## Electronic supplementary material

Below is the link to the electronic supplementary material.

Supplementary material 1 (DOCX 17 KB)
