# Supplementary material for: Utility scores for different health states related to depression: individual participant data analysis
Source: Qual Life Res. 2017 Mar 4;26(7):1649–58. doi: 10.1007/s11136-017-1536-2 (PMC5486895; doi:10.1007/s11136-017-1536-2)
Supplement: Supplementary file 1 — Supplementary material 1 (DOCX 17 KB) [file 11136_2017_1536_MOESM1_ESM.docx]

**Supplementary material**

**S1.** Table presenting the baseline model and the adjusted model including the random coefficients of covariates

| **Health State** | **Baseline model** |  | **Crude model^a^** |  | **Adjusted model^b^** |  |
| --- | --- | --- | --- | --- | --- | --- |
| **EQ-5D (N_obs_ = 4979)** | ***B* (SE)** | **95% CI** | ***B* (SE)** | **95% CI** | ***B* (SE)** | **95% CI** |
| Remission (*intercept*) | 0.75 (0.01) | 0.73 to 0.77 | 0.72 (0.01) | 0.70 to 0.74 | 0.70 (0.02) | 0.67 to 0.73 |
| Minor depression | -0.11 (0.01) | -0.13 to -0.09 | -0.08 (0.01) | -0.11 to -0.06 | -0.08 (0.01) | -0.10 to -0.06 |
| Mild depression | -0.18 (0.01) | -0.22 to -0.17 | -0.12 (0.01) | -0.15 to -0.10 | -0.13 (0.01) | -0.15 to -0.10 |
| Moderate depression | -0.20 (0.01) | -0.22 to -0.17 | -0.17 (0.01) | -0.19 to -0.14 | -0.18 (0.01) | -0.20 to -0.15 |
| Severe depression | -0.41 (0.02) | -0.45 to -0.38 | -0.33 (0.02) | -0.36 to -0.29 | -0.34 (0.02) | -0.37 to -0.30 |
| Comorbidity (yes) | N/A | N/A | N/A | N/A | 0.04 (0.01) | 0.01 to 0.07 |
| Gender (female) | N/A | N/A | N/A | N/A | -0.02 (0.01) | -0.05 to 0.01 |
| Civil status (married/living together) | N/A | N/A | N/A | N/A | 0.04 (0.01) | 0.02 to 0.07 |
| Randomization (intervention) | N/A | N/A | N/A | N/A | 0.01 (0.01) | -0.01 to 0.03 |
| Age | N/A | N/A | N/A | N/A | 0.00 (0.00) | -0.01 to 0.03 |
| Intermediate education (vs low) | N/A | N/A | N/A | N/A | 0.03 (0.01) | 0.01 to 0.05 |
| High education (vs low) | N/A | N/A | N/A | N/A | 0.05 (0.01) | 0.03 to 0.07 |
| **SF-6D (N_obs_ = 1726)** |  |  |  |  |  |  |
| Remission (*intercept*) | 0.71 (0.01) | 0.69 to 0.73 | 0.69(0.01) | 0.67 to 0.71 | 0.69 (0.01) | 0.67 to 0.71 |
| Minor depression | -0.08 (0.01) | -0.10 to -0.06 | -0.07 (0.01) | -0.09 to -0.05 | -0.06 (0.01) | -0.08 to -0.04 |
| Mild depression | -0.13 (0.01) | -0.15 to -0.11 | -0.10 (0.01) | -0.12 to -0.08 | -0.10 (0.01) | -0.12 to -0.08 |
| Moderate depression | -0.15 (0.01) | -0.17 to -0.13 | -0.13 (0.01) | -0.15 to -0.11 | -0.13 (0.01) | -0.15 to -0.11 |
| Severe depression | -0.16 (0.01) | -0.18 to -0.14 | -0.14 (0.01) | -0.16 to -0.12 | -0.14 (0.01) | -0.15 to -0.11 |
| Comorbidity (yes) | N/A | N/A | N/A | N/A | -0.06 (0.01) | -0.08 to -0.05 |
| Gender (female) | N/A | N/A | N/A | N/A | 0.01 (0.01) | -0.01 to 0.03 |
| Civil status (married/living together) | N/A | N/A | N/A | N/A | 0.01 (0.01) | -0.01 to 0.03 |
| Randomization (intervention) | N/A | N/A | N/A | N/A | 0.00 (0.01) | -0.02 to 0.02 |
| Age | N/A | N/A | N/A | N/A | 0.00 (0.00) | -0.02 to 0.02 |
| Intermediate education (vs low) | N/A | N/A | N/A | N/A | 0.01 (0.01) | -0.01 to 0.03 |
| High education (vs low) | N/A | N/A | N/A | N/A | 0.01 (0.01) | -0.01 to 0.03 |

^a^ Model is controlled for the hierarchical structure of the data

**^b^** Model is controlled for covariates

CI= Confidence intervals; N_obs_= Number of observations; SE= Standard error
